# Low, but Not High, Pulsating Fluid Shear Stress Affects Matrix Extracellular Phosphoglycoprotein Expression, Mainly via Integrin β Subunits in Pre-Osteoblasts

**DOI:** 10.3390/cimb46110738

**Published:** 2024-11-04

**Authors:** Jianfeng Jin, Behrouz Zandieh-Doulabi

**Affiliations:** Department of Oral Cell Biology, Academic Centre for Dentistry Amsterdam (ACTA), University of Amsterdam and Vrije Universiteit Amsterdam, Amsterdam Movement Sciences, 1081 LA Amsterdam, The Netherlands; bzandiehdoulabi@acta.nl

**Keywords:** integrin α/β subunit, matrix extracellular phosphoglycoprotein, mechanical loading, osteogenic differentiation, pre-osteoblasts, pulsating fluid shear stress

## Abstract

Matrix extracellular phosphoglycoprotein (Mepe), present in bone and dentin, plays important multifunctional roles in cell signaling, bone mineralization, and phosphate homeostasis. Mepe expression in bone cells changes in response to pulsating fluid shear stress (PFSS), which is transmitted into cells through integrin-based adhesion sites, i.e., α and β subunits. Whether and to what extent PFSS influences Mepe expression through the modulation of integrin α and/or β subunit expression in pre-osteoblasts is uncertain. Therefore, we aimed to test whether low and/or high PFSS affects Mepe expression via modulation of integrin α and/or β subunit expression. MC3T3-E1 pre-osteoblasts were treated with ± 1 h PFSS (magnitude: 0.3 Pa (low PFSS) or 0.7 Pa (high PFSS); frequency: 1 Hz). Single integrin fluorescence intensity in pre-osteoblasts was increased, but single integrin area was decreased by low and high PFSS. Expression of two integrin α subunit-related genes (*Itga1* and *Itga5 2*) was increased by low PFSS, and one (*Itga5 2*) by high PFSS. Expression of five integrin β subunit genes (*Itgb1*, *Itgb3*, *Itgb5*, *Itgb5 13*, and *Itgb5 123*) was increased by low PFSS, and three (*Itgb5*, *Itgb5 13*, and *Itgb5 123*) by high PFSS. Interestingly, *Mepe* expression in pre-osteoblasts was only modulated by low, but not high, PFSS. In conclusion, both low and high PFSS affected integrin α and β subunit expression in pre-osteoblasts, while integrin β subunit expression was more altered by low PFSS. Importantly, *Mepe* gene expression was only affected by low PFSS. These results might explain the different ways that *Mepe*-induced changes in pre-osteoblast mechanosensitivity may drive signaling pathways of bone cell function at low or high impact loading. These findings might have physiological and biomedical implications and require future research specifically addressing the precise role of integrin α or β subunits and *Mepe* during dynamic loading in bone health and disease.

## 1. Introduction

Matrix extracellular phosphoglycoprotein (Mepe, also called osteoblast/osteocyte factor 45) was first isolated from tumors associated with hypophosphatemic osteomalacia [1,2]. Mepe is a member of the small integrin-binding ligand N-glycosylated (SIBLING) family of extracellular matrix proteins, and is similar to other SIBLING proteins (e.g., osteopontin (OPN), dentin matrix protein 1 (DMP-1), bone sialoprotein (BSP), and dentin sialophosphoprotein (DSPP)). Mepe is thought to have multiple functions, including cell signaling, mineralization, and mineral homeostasis [3]. However, the exact role of Mepe in the regulation of mineralization and bone formation as well as the underlying mechanisms are still unclear. Some studies have revealed a negative effect of Mepe on mineralization [4,5,6], whereas others have claimed that Mepe has a positive effect on mineralization [7]. Ablation of Mepe in mice leads to the enhancement of bone formation (negative effect), indicating that Mepe is an inhibitor of mineralization in bone [8,9]. On the other hand, Mepe is expressed in bone during the proliferation and early-maturation phases by fully differentiated osteoblasts, with maximal expression during mineralization (positive effect) [10]. In addition, the mid-terminal fragment of Mepe has been reported to increase the proliferation of human bone marrow stromal cells and to promote new bone formation [11] and dental pulp repair [12,13]. Moreover, Mepe might be a soluble factor produced after mechanical loading of osteocytes, the key regulators of osteoclast formation and bone resorption, leading to the inhibition of osteoclastogenesis [5]. This leads to the next question: What is the relationship between Mepe and mechanical loading? Four-point bending has been shown to increase Mepe gene expression in the tibia of female Wistar rats, which might regulate bone mineralization and phosphate homeostasis [14]. Increased understanding of the communication between mechanically loaded osteocytes or osteoblasts and osteoclasts will help to clarify the function of Mepe in bone cells, especially under an environment of mechanical loading.

The bones in the human body are constantly subjected to mechanical forces, which are essential for the growth, development, and maintenance of bone [15]. These forces are sensed by bone cells, such as osteoblasts, which build new bone and ensure that bones grow correctly and remain strong [15]. Regular physical exercise such as running, cycling, or various other sports provide mechanical stimuli to the bone and help to maintain healthy and strong bone. In contrast, mechanical unloading conditions, such as long-term bed rest or space traveling (in the case of astronauts), cause severe bone loss [16,17]. Mechanical loading at a magnitude in the physiological range has an anabolic effect on bone mass, but in the supraphysiological range it causes bone loss [18,19,20]. Therefore, reasonable exercise at optimal force rate or magnitude is beneficial for bone adaptation [21]. Walking has little effect on bone formation [22], while jogging results in a small increase in lumbar spine bone mineral density [23]. Lower intensity endurance exercise has less effect than strengthening and weight-bearing exercise on bone formation [24]. However, the molecular mechanism by which mechanical forces are converted into a bone anabolic response, i.e., osteogenesis or bone formation, still needs to be further unraveled (e.g., force rate or magnitude) in (pre)osteoblasts [21].

**Figure 1:**
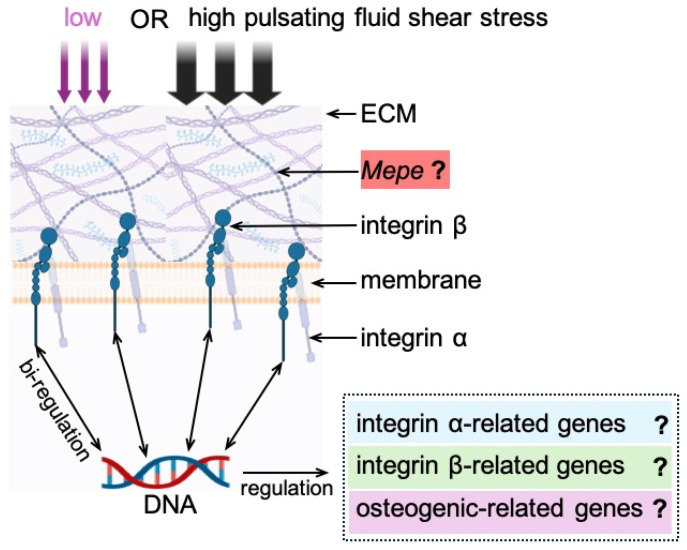
Schematic diagram illustrating PFSS-induced changes in Mepe, and integrin subunit-related genes. ECM: extracellular matrix; *Mepe*: matrix extracellular phosphoglycoprotein; PFSS: pulsating fluid shear stress.

Osteoblasts sense mechanical forces and translate them into biochemical signals to regulate cell shape and function via the extracellular matrix, gap junctions, integrins, ion channels, focal adhesion kinases, and the cytoskeleton (e.g., actin filaments, microtubules, and intermediate filaments) [25,26]. Integrins, as bridges, mediate mechanical signals between the ECM and the cytoskeleton via focal adhesion kinases, are able to sense matrix features, e.g., texture, stiffness and external forces, and transfer these signals into biochemical or biological signals [27,28]. This transduction process is called “mechanical signal transduction” [27]. When integrins are active, they recruit different integrin associated proteins (IAP) near the cell membrane, e.g., talin, vinculin, and kindlin. The IAPs enhance the number of specific cell surface receptors, which have a similar function to integrins and involve amplifying effects, such as the opening of mechanically gated channels (e.g., PIEZO1 channels) [29,30]. IAPs convert the signal received by integrins to the cytoskeleton (e.g., actin filaments), which undergo contraction, changing the tension of the phospholipid bilayer on the membrane, thereby leading to the opening of mechanically gated channels [27,29,30]. Mechanical force also induces substantial changes in osteoblast cell body and nucleus volume, which are accompanied by changes in integrin and paxillin [26]. Integrins emerge as cell adhesion receptors, which mediate the interaction between cells and the extracellular matrix and act as mechanotransducers, regulating osteogenesis [31,32]. MC3T3-E1 pre-osteoblast proliferation is decreased when the integrin gene is knocked out. Furthermore, integrins (e.g., β1 integrin) increase osteogenic differentiation and ECM production by mechanically loaded osteoblasts, indicating that integrins are important for ECM integrity, biomechanical properties, and bone quality in vivo [33]. Based on the different integrin binding characteristics, integrins are divided into four types, i.e., leukocyte cell-adhesion integrins (α4β1, α9β1), laminin-binding integrins (α1β1, α3β1), collagen (GROGER)-binding integrins (α1β1, α2β1), and RGD-binding integrins (α5β1, αvβ3) [34]. These integrin isoforms are tissue-based (or specific) and developmentally regulated [35]. Their properties are still not as well understood as those of the α and β subunits. Importantly, integrins interact with extracellular matrix proteins, e.g., osteopontin, fibronectin, fibrinogen, and vitronectin, which highly likely contain small peptide sequences (ARG-Gly-Asp (RGD)) as integrin recognition motifs [35,36]. These motifs promote cells to adhere to the extracellular matrix and convert mechanical force signals into biochemical signals in the cells via integrin α and β subunits [37]. However, little is known about how *Mepe* expression is associated with changes in integrin α and/or β subunit expression in response to mechanical loading of different magnitudes in bone cells. More specifically, whether, and to what extent, pulsating fluid shear stress (PFSS) influences *Mepe* expression through the modulation of integrin α and/or β subunit expression in osteoblasts is uncertain (Figure 1).

In this study, we aimed to test whether low and/or high PFSS affects *Mepe* expression via modulation of integrin α and/or β subunit expression in MC3T3-E1 pre-osteoblasts. We focused on the morphology of pre-osteoblasts and structure of integrin (fluorescence intensity per single integrin and area per single integrin) subjected to low or high PFSS. We analyzed gene expression related to integrin α subunit, β subunit, and osteogenic differentiation in pre-osteoblasts treated with low or high PFSS.

## 2. Materials and Methods

### 2.1. Cell Culture

MC3T3-E1 pre-osteoblasts were cultured in 75 cm^2^ flasks (Thermo Fisher Scientific, Waltham, MA, USA) in α–minimal essential medium (α-MEM, Gibco, Paisley, UK), supplemented with 10% fetal bovine serum (FBS; Gibco), 300 μg/mL penicillin (Sigma-Aldrich, St. Louis, MI, USA), 250 μg/mL streptomycin (Sigma-Aldrich), and 1.25 μg/mL fungizone (Gibco), in a humidified atmosphere of 5% CO_2_ in air at 37 °C. The medium was exchanged every 72 h. Upon reaching 80–90% confluence, cells were harvested using 0.5 mM ethylenediaminetetraacetic acid (EDTA) and 0.25% trypsin (Gibco) for around 3 min at 37 °C, replated at 1.5 × 10^5^ cells per 75 cm^2^ flask (Greiner Bio-One, Kremsmuenster, Austria), and passaged until the cells reached 80–90% confluence again.

### 2.2. Low- and High-PFSS Treatment of Cells

One day before mechanical loading by PFSS, MC3T3-E1 pre-osteoblasts were seeded at 1 × 10^3^ cells/cm^2^ (small glass slide: 24 × 24 × 0.15 mm (length × width × height)) or 3 × 10^3^ cells/cm^2^ (big glass slide: 36 × 76 × 1 mm (length × width × height)) on poly-L-lysine-coated (50 μg/mL; poly-L-lysine hydrobromide; Sigma-Aldrich) small and big glass slides. A “small chamber” (14 × 14 × 0.2 mm (length × width × height), inner dimensions) was used for integrin observation by confocal microscopy. A “big chamber” (58 × 32 × 0.3 mm (length × width × height), inner dimensions) was used for observing cell morphology, and measuring gene expression. In both chambers, cells were treated with low PFSS (magnitude: 0.3 Pa) or high PFSS (magnitude: 0.7 Pa) at 1 Hz for 1 h at 37 °C. The PFSS magnitude was calculated as follows [38].

τ = 6·Q·µ/(b·h^2^)

τ: shear stress magnitude; Q: flow rate (low PFSS: 7 mL/min; high PFSS: 20 mL/min); µ: viscosity (0.0078 dynes.sec/cm^2^); b: slit width; h: channel height.

Static control cultures were kept in a Petri dish under similar conditions as experimental cultures, i.e., α-MEM with 10% FBS, 300 μg/mL penicillin, 250 μg/mL streptomycin, and 1.25 μg/mL fungizone, as well as 1 h incubation at 37 °C.

### 2.3. Cell Morphology

Before and after low- or high-PFSS treatment, the morphology of cells on the glass slides was observed by light microscopy (Leica, Wetzlar, Germany). Three pictures were taken randomly from each glass slide under 10× magnification. Then, the pictures were used to assess cell morphology of cells treated without/with low or high PFSS.

### 2.4. Integrin Structure

Cells treated with 1 h low or high PFSS were fixed with 4% paraformaldehyde (Merck, Rahway, NJ, USA) in phosphate-buffered saline (PBS; Gibco) for 15 min at 37 °C, and permeabilized with 0.2% Triton X-100 (Serva Electrophoresis GmbH, Heidelberg, Germany) in PBS at room temperature for 10 min. After washing 3 times with PBS, the cells were blocked with 5% bovine serum albumin (BSA) for 1 h at room temperature. Then the cells were incubated with primary antibody against integrin by using rabbit recombinant monoclonal integrin α5 antibody (dilution 1:100; Abcam, Cambridgeshire, UK) at 4 °C overnight. The next day, cells were washed 3 times with PBS, and incubated with Alexa Fluor 488 goat-anti-rabbit antibody (dilution 1:500; Thermo Fisher Scientific, Waltham, MA, USA) for 1 h at room temperature in the dark. After washing 3 times with PBS, the cells were incubated with 40,6-diamidine-20-phenylindole dihydrochloride (DAPI; Merck, Whitehouse Station, NJ, USA) for 10 min at room temperature in the dark. After washing 3 times with PBS, the cells were mounted in the Vecta-shield device (Vector Laboratories, Burlingame, CA, USA) for laser scanning confocal microscopy to assess integrin structure in pre-osteoblasts without/with low or high PFSS.

### 2.5. Gene Expression Analysis

Total RNA was isolated from cells treated without/with 1 h low or high PFSS by TRIzol^®^ reagent (Life Technologies, Carlsbad, CA, USA), and stored at −80 °C prior to further use. Complementary DNA (cDNA) synthesis was performed according to the First Strand cDNA Synthesis kit (Thermo Fisher Scientific, Waltham, MA, USA) in a thermocycler GeneAmp^®^ System 9700 PE (Applied Biosystems, Waltham, MA, USA). cDNA was stored at −20 °C prior to RT-PCR analysis, and diluted 5× for gene expression analysis. RT-PCR reactions were performed using 1 µL cDNA per reaction and LightCycler^®^ 480 SYBR^®^ Green I Mastermix (Roche Diagnostics, Basel, Switzerland) in a LightCycler^®^ 480 (Roche Diagnostics, Basel, Switzerland). RT-PCR conditions for all genes were 10 min pre-incubation at 95 °C, followed by 45 cycles of amplification at 95 °C for 10 s, 56 °C for 5 s, 72 °C for 10 s, and 78 °C for 5 s. Melting curve analysis was then performed with LightCycler^®^ software (version 1.2, Roche Diagnostics); crossing points were assessed and plotted versus the serial dilution of known concentrations of the internal standard. For gene expression analysis, the values of target gene expression were normalized using *Pbgd* (Forward primer sequence (5′-3′) (Forward): AGTGATGAAAGATGGGCAACT; Reverse primer sequence (5′-3′) (Reverse): TCTGGACCATCTTCTTGCTGA) to obtain relative gene expression. RT-PCR was used to assess expression of the integrin α subunit-related genes (*Itga1* (Forward: AATGTCAGCCTCACCGTCAA; Reverse: AGTTAACCACGTCTCCTGTC), *Itga3* (Forward: °TCGGCAGACTGAGCGACAAC; Reverse: GTCACTCCAAGCCACATATCC), *Itga5 1* (Forward: CATGAAGGCAGGCACCAGTCT; Reverse: CTGAGGCTGGTCTTGAGGATT), and *Itga5 2* (Forward: TAAGTGGCCGGTTGCCTGAGTT; Reverse: AGACAGCACCACCTTGCAGTA)), integrin β subunit-related genes (*Itgb1* (Forward: ACTGGCAGTGCATGTGACTGT; Reverse: AGACGCCAAGGCAGGTCTGA), *Itgb3* (Forward: GTGGTCCTGCTGTCAGTGATGG; Reverse: CCGGTAGGTGATATTGGTGAAG), *Itgb5* (Forward: GTGGGTAGACACATCGTCAAAG; Reverse: TGGGCAGTTCTGTGTAGCTGAA), *Itgb5 12* (Forward: CCAGATGACGCCGCAGGAGAT; Reverse: CCAGGCTCCGGATGTTCTCCAA), *Itgb5 13* (Forward: CAGGGCCCGTTATGAAATG; Reverse: CATTATCCGTGCGTGCCTA), and *Itgb5 123* (Forward: CTCTGCACTTGCTGGTGTTCA; Reverse: CAAGCAAGGCAAGCGATGGAT)), and osteogenesis-related genes (*Ki67* (Forward: CCCTCAGCAAGCTGAGAA; Reverse: AGAGGCGTATTAGGAGGCAAG), *Bmp2* (Forward: CATCCAGCCGACCCTTGT; Reverse: GAGTGCCTGCGGTACAGATCT), *Runx2* (Forward: ATGCTTCATTCGCCTCAC; Reverse: ACTGCTTGCAGCCTTAAAT), *Fgf2* (Forward: GGCTTCTTCCTGCGCATCCA; Reverse: TCCGTGACCGGTAAGTATTG), *Cox2* (Forward: TTGCTGTTCCAATCCATGTCA; Reverse: GGTGGGCTTCAGCAGTAATTTG), *Dmp1* (Forward: CGGCTGGTGGACTCTCTAAG; Reverse: CGGGGTCGTCGCTCTGCATC), *Mepe* (Forward: GGAGCACTCACTACCTGAC; Reverse: TAGGCACTGCCACCATGT), and *Sost* (Forward: GTGCCTCATCTGCCTACTTGTG; Reverse: CCGCCCGGTTCATGGT)). Four independent experiments were performed (n = 4).

### 2.6. Statistical Analysis

All data are expressed as mean ± SD from at least three independent, separate experiments (cell morphology: 3 independent experiments with 9 glass slides (n = 3); integrin structure: 3 independent experiments with 9 glass slides (n = 3); integrin number, fluorescence, and area: 15 pictures from 3 independent experiments with 9 glass slides (n = 3); gene expression: 4 independent experiments with 12 glass slides (n = 4)). Differences were tested with one-way analysis of variance combined with Tukey according to the following experimental design. There was no matching or pairing. A Gaussian distribution of residuals was assumed, allowing the use of ANOVA. Equal SDs were assumed, allowing the use of an ordinary ANOVA test. There were no repeated measures, and multiple comparisons were performed in follow-up tests comparing the mean of each column with the mean of every other column. Differences were considered significant if *p* < 0.05. Statistical analysis was performed using GraphPad Prism^®^ 8.0 (GraphPad Software Inc.; Boston, MA, USA).

## 3. Results

### 3.1. Cell Morphology Without/with Low or High PFSS

**Figure 2:**
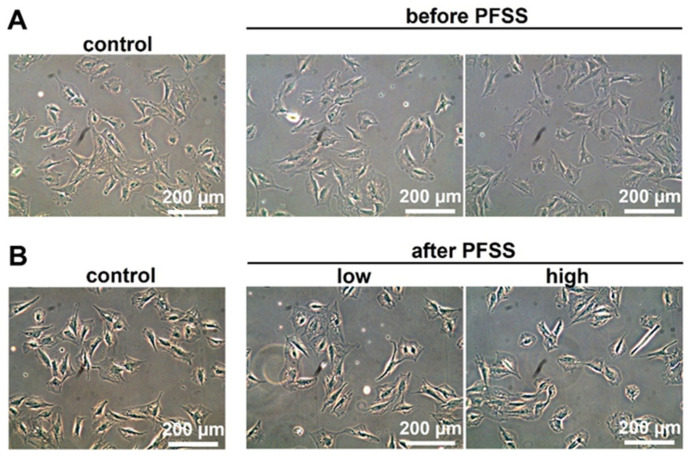
Effect of low and high PFSS on pre-osteoblast morphology as observed by light microscopy. (**A**) Cell morphology before PFSS; (**B**) cell morphology after PFSS. Scale bar: 200 µm. PFSS: pulsating fluid shear stress.

Before mechanical loading, the cell morphology on the glass slide surface was similar in all cell cultures (Figure 2A). All cells spread well and had an irregular shape (e.g., spindle shape, ellipse, or polygon, etc.). After 1 h without or with mechanical loading by low or high PFSS, the cell morphology was similar to that before mechanical loading in the control cells (Figure 2B).

### 3.2. Integrin Structure in Cells Without/with Low or High PFSS

**Figure 3:**
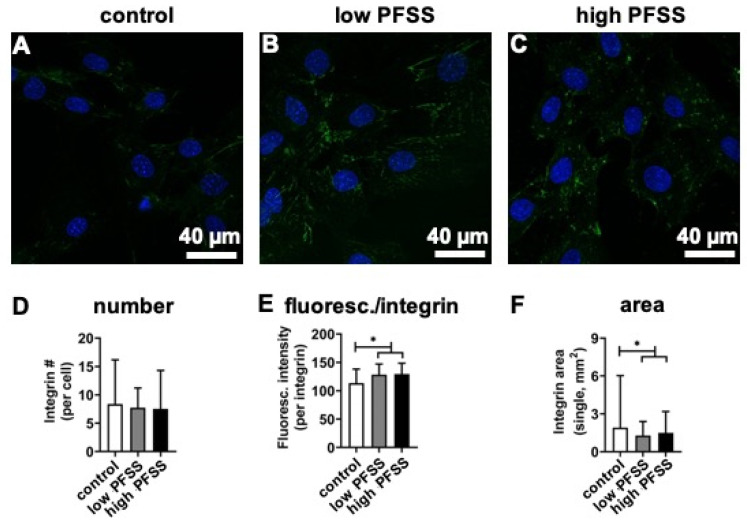
Effects of low and high PFSS on integrin α5 in pre-osteoblasts by laser scanning electronic microscopy. (**A**–**C**) Integrin structure without/with low/high PFSS. (**D**) Quantification of integrin number. (**E**) Quantification of integrin fluorescence per integrin. (**F**) Quantification of single integrin area. Green: integrins, blue: nuclei. PFSS: pulsating fluid shear stress. Scale bar: 40 µm. * *p* < 0.05.

Integrins (green color) were almost not visible in the control cells without mechanical loading (Figure 3A). A visible increase in green color intensity (integrins) was observed in low-PFSS and high-PFSS-treated cells (Figure 3B,C). The number of integrins with low and high PFSS was similar in the control (Figure 3D). However, low and high PFSS increased the fluorescence intensity of each integrin, i.e., integrin α5 (Figure 3E). Furthermore, low and high PFSS decreased the area of each integrin (Figure 3F).

### 3.3. Integrin α and β Subunit-Related Gene Expression in Cells Without/with Low or High PFSS

**Figure 4:**
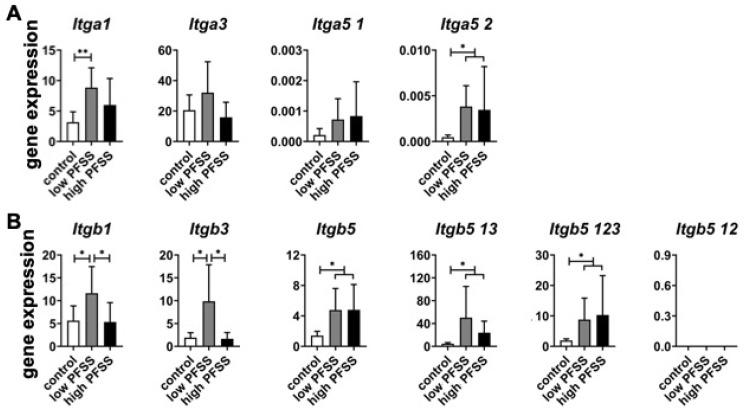
Effects of low and high PFSS on integrin α and β subunit-related gene expression in pre-osteoblasts. Gene expression related to (**A**) integrin α subunits, (**B**) integrin β subunits. PFSS: pulsating fluid shear stress. * *p* < 0.05, **p < 0.01.

Analysis of integrin α subunit gene expression (Figure 4A) revealed that low PFSS significantly increased expression of *Itga1* (2.8-fold) and *Itga5 2* (8.0-fold). High PFSS significantly enhanced expression of *Itga5 2* (7.3-fold). Both low and high PFSS did not affect *Itga3* and *Itga5 1* expression (Figure 4A).

Analysis of integrin β subunit gene expression (Figure 4B) showed that low PFSS significantly increased expression of *Itgb1* (2.1-fold), *Itgb3* (5.2-fold), *Itgb5* (3.4-fold), *Itgb5 13* (10.8-fold), and *Itgb5 123* (4.6-fold). High PFSS significantly enhanced expression of *Itgb5* (3.4-fold), *Itgb5 13* (5.2-fold), and *Itgb5 123* (5.3-fold), but not *Itgb1* and *Itgb3* expression. *Itgb5 12* expression was undetectable (Figure 4B).

### 3.4. Osteogenic Differentiation-Related Gene Expression in Cells Without/with Low or High PFSS

**Figure 5:**
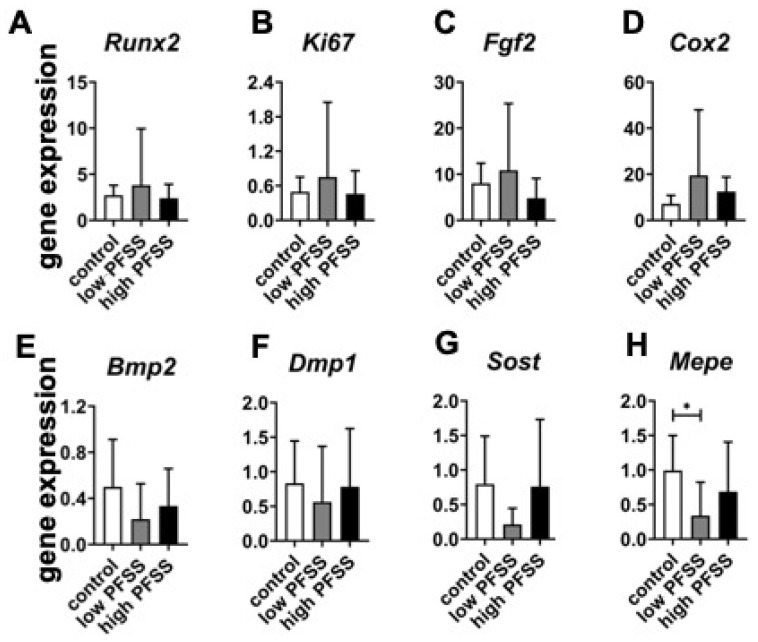
Effects of low and high PFSS on osteogenic gene expression in pre-osteoblasts. Gene expression of (**A**) Runx2, (**B**) Ki67, (**C**) Bmp2, (**D**) Fgf2, (**E**) Cox2, (**F**) Dmp1, (**G**) Mepe, and (**H**) Sost without/with low/high PFSS. PFSS: pulsating fluid shear stress. * *p* < 0.05.

One-hour low and high PFSS did not affect *Runx2* expression (Figure 5A). Expression levels of *Ki67*, *Fgf2*, and *Cox2* showed a similar trend, i.e., they were also not affected by low or high PFSS (Figure 5B–E). *Bmp2*, *Dmp1*, and *Sost* genes displayed similar expression levels, and were not affected by low or high PFSS (Figure 5E–G). Interestingly, low but not high PFSS significantly decreased *Mepe* expression (0.3-fold; Figure 5H).

## 4. Discussion

This study aimed to test whether low and/or high PFSS affects *Mepe* expression via modulation of integrin α and/or β subunit expression in MC3T3-E1 pre-osteoblasts. Our findings were as follows: (i) Cell morphology was not affected by low or high PFSS. (ii) Single integrin fluorescence intensity and area were changed by low and high PFSS. (iii) The expression of two integrin α subunit-related genes (*Itga1* and *Itga5 2*) was increased by low PFSS, and expression of one integrin α subunit-related gene (*Itga5 2*) was enhanced by high PFSS. (iv) The expression of five integrin β subunit genes (*Itgb1*, *Itgb3*, *Itgb5*, *Itgb5 13*, and *Itgb5 123*) was increased by low PFSS, and expression of three integrin β subunit genes (*Itgb5*, *Itgb5 13*, and *Itgb5 123*) was increased by high PFSS. (v) High PFSS did not affect any of eight osteogenesis-related genes. (vi) Low, but not high, PFSS decreased *Mepe* expression.

Pre-osteoblasts were treated for 1 h with 0.3 Pa (low PFSS) or 0.7 Pa (high PFSS), at 1 Hz frequency. Both low and high PFSS were used to treat the cells, since we found earlier that the response of pre-osteoblasts is directly proportional to the rate of PFSS, which depends on the amplitude and frequency of stress [39,40]. High PFSS affects cell body volume, nuclear volume, and paxillin expression in pre-osteoblasts [41]. Moreover, low PFSS affects cytoskeleton-related gene expression in these cells (unpublished data). Moreover, 1 h PFSS was applied since this is long enough to allow maximal activation production of signaling molecules, such as nitric oxide and prostaglandins, which are known parameters for bone cell activation as they are early mediators of mechanical loading-induced bone formation [38,41]. Therefore, low and high PFSS in the physiological range can affect pre-osteoblast behavior. In vivo, shear stress induced by interstitial fluid through bone cell surface is a likely signal for cell adaptive responses [38]. Furthermore, the rate (determined by magnitude and frequency) of mechanical loading determines bone quality [42]. Nitric oxide production is linearly dependent on the rate of mechanical loading, which is a parameter for bone cell activation [38]. It might explain why low and high PFSS affect pre-osteoblasts differently. Additionally, alterations in osteoblast cytoskeletal structure in response to shear stress occur within minutes [26,41,43,44]. Therefore, we chose 1 h PFF as an end point for our investigations. 

The observed morphology of pre-osteoblasts seeded on glass slides indicated that the cells spread well. One-hour low or high PFSS did not change the cell morphology, as observed from a top view using light microscopy at the cell (or micro) scale. These bone cells sense and transmit (called mechanotransduction) [41] the physical signals to the inside of the cells, and to adjacent cells. This process of mechanotransduction in bone cells might change the cell structure (at the nanoscale), but not the cell morphology (at the microscale). These data in bone cells are different to earlier observations in muscle cells by Haroon et al., where PFSS did modulate the morphology and number of muscle stem cells [45]. This difference in results between the current study and the study by Haroon et al. lies in the types of cells, pre-osteoblasts and muscle cells. Pre-osteoblasts are much flatter than muscle stem cells, and adhere more strongly to the glass slide. As a result, changes in cell morphology and number might depend on cell type. Additionally, it has been shown that mouse long bone cells subjected to lower streaming potentials and chemotransport, but the same high shear stress, exhibit a similar response, as revealed by the release of nitric oxide production and prostaglandin E2 in the flow medium [38]. Moreover, nitric oxide production induced by high PFSS is accompanied by parallel alignment of actin stress fibers in osteoblasts [44]. Prostaglandin E2 induced by high PFSS is related to loading stimulation of focal adhesions which are formed after disrupting the cytoskeleton in osteoblasts, indicating that the cytoskeleton (or cell morphology) is related to the release of nitric oxide production and prostaglandin E2 induced by mechanical loading [44]. In this study, cell morphology was not affected by low and high PFSS, which is not consistent with the findings by McGarry’s et al. [44]. However, this study is consistent with our previous findings, showing that high PFSS does not affect filament actin and microtubules [26,41].

Integrins connecting the extracellular matrix with the cytoskeleton recruit many proteins to their short cytoplasmic tails upon ligand binding, thereby assembling a variety of adhesion structures which differ in subcellular localization and morphology, as well as in protein composition and mechanical properties [46,47]. Previously we have shown that high PFSS increases integrin fluorescence, number, area, and size in MC3T3-E1 pre-osteoblasts [41]. In the current study, low and high PFSS increased integrin fluorescence per integrin and decreased single integrin area, but did not affect integrin number. This might be explained by a difference in integrin staining intensity. In our previous study, we used α5 rat monoclonal IgG-2a (Abcam) for integrin staining, while this study used rabbit recombinant monoclonal integrin α5 antibody (Abcam), since α5 rat monoclonal IgG-2a could not be purchased anymore. Confocal microscopy revealed that there was no significant difference in fluorescence intensity or area between low- and high-PFSS-treated cells. This might be explained by the fact that we have tested the effect of PFSS of two single magnitudes within the physiological range, but not PFSS resembling disuse or overuse, since they cause bone cell apoptosis and cell death [48]. The physical signals are sensed by the cells from the extra cellular matrix into the cytoskeleton. Additionally, with respect to the decreased single integrin area, the height of integrins might be increased by low and high PFSS.

The α subunit of each integrin is the primary determinant of its extracellular ligand specificity, modulating the formation of intracellular adhesion complexes, and regulating downstream signaling [49,50]. In this study, four types of integrin α subunit-related genes (*Itga1*, *Itga3*, *Itag5 1*, and *Itga5 2*) were investigated. *Itga1* and *Itga5 2* were enhanced by low PFSS, indicating that the signal of low PFSS might be sensed mainly via integrin α1 and α5 2 subunits. *Itga5 2* was also increased by high PFSS, showing that the signal of high PFSS might be sensed mainly via the integrin α5 2 subunit. The expression of two types of integrin α5 subunits were investigated in this study, i.e., Itga5 1 and Itga5 2. Integrin α5 deficiency hinders load-induced connexin 43 hemichannel opening and release of prostaglandin E2, thereby attenuating the effects of loading on the reduction in sclerostin and the increase in β catenin [51]. We found that only integrin α5 2 was affected by low PFSS, revealing that connexin 43 might be regulated only by integrin α5 2 during low PFSS treatment. Future work should further investigate connexin 43 and integrin α5 under environments of low or high PFSS.

The integrin β subunit binds acidic residues in intercellular adhesion molecules and in cytoplasmic adapters, e.g., paxillin, talin, and kindlin, to facilitate cell adhesion with the extracellular matrix [49]. Integrins interact with the actin cytoskeleton via the talin- and kindlin-binding motifs present in the cytoplasmic domains of their β subunits [49,52]. In this study, five integrin β subunit-related genes (*Itgb1*, *Itgb3*, *Itgb5*, *Itgb5 13*, and *Itgb5 123*) were enhanced by low PFSS, indicating that the signal of low PFSS might be sensed mainly via the integrin β1, β3, β5, β5 13, and β5 123 subunits. Three integrin β subunit-related genes (*Itgb5*, *Itgb5 13*, and *Itgb5 123*) were increased by high PFSS, showing that the signal of high PFSS might be sensed mainly via the integrin β5, β5 13, and β5 123 subunits. The integrin β5 12 subunit-related gene was not detectable. In mammals, each integrin is composed of an α and β subunit in a noncovalent complex. Eighteen α subunits and eight β subunits generate 24 unique heterodimeric transmembrane receptors, excluding spice- and glycosylation variants [53]. Each α and β subunit contains a short cytosolic tail, single-span helical transmembrane domain, and large ectodomain, except the β4 subunit [54]. Most α subunits only form one kind of complex with one β subunit. However, the α4 and αv subunits interact with more than one β subunit, e.g., α4β1, α4β7, αvβ1, αvβ3, αvβ5, αvβ6, and αvβ8 [35]. The β1 subunit can form heterodimeric complexes with 12 α subunits, but β4, β5, β6, and β8 only interact with one α subunit [35]. In the future, different sequences of this gene should be designed and tested to confirm the lack of expression of this gene. Our study investigated different types of integrins. The heterogeneity of integrin mechanical properties has been shown to determine the response of osteoblasts to mechanical loading [55]. Thus measurement of different integrin dimers is highly important for a better understanding of mechanotransduction dynamics [55]. Additionally, integrin α5β1-related gene expression is enhanced by dynamic loading [56]. However, in our study, we did not investigate the combined integrin α- and β-related genes under low or high PFSS. Therefore, future studies should also address other types of integrin and the combined integrin α and β, with or without low or high PFSS. A possible mechanism by which low and high PFSS differentially affect integrin α- and β-related gene expression might be related to the opening of ion channels (e.g., calcium, and PIEZO1) on the cell membrane. Integrins are located on the cell membrane as well. Since the magnitude of low and high PFSS is different, the cells will receive different mechanical signals, possibly resulting in opening or closing of ion channels.

**Figure A1:**
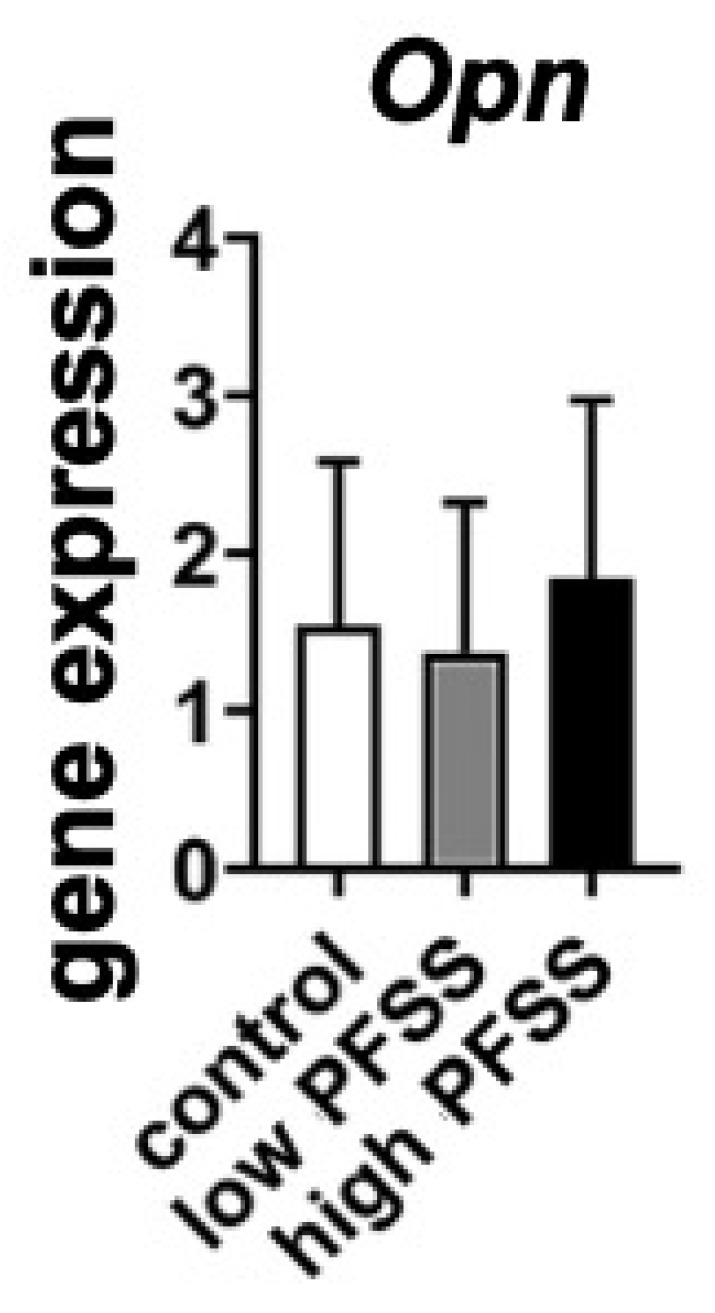
Effects of low and high PFSS on osteopontin (Opn) gene expression in pre-osteoblasts. PFSS: pulsating fluid shear stress. Primer sequence: Forward: CCCGGTGAAAGTGACTGATT; Reverse: TTCTTCAGAGGACACAGCATTC.

The initiation of osteogenesis primarily occurs as mesenchymal stem cells undergo differentiation into osteoblasts. This differentiation process is important for bone formation and homeostasis and is regulated by two intricate processes, i.e., cell signal transduction and transcriptional gene expression [57]. In this study, analysis of the expression of genes related to osteogenic differentiation was performed. Seven genes (i.e., *Runx2*, *Ki67*, *Fgf2*, *Cox2*, *Bmp2*, *Dmp1*, and *Sost*) were not affected by low and high PFSS, which corresponds to our previous findings, showing that 1 h of PFSS treatment did not affect osteogenic differentiation of pre-osteoblasts [26]. These data met our expectations. However, one-hour PFSS affects mitochondrial biogenetic-related *Pgc-1α* gene expression (unpublished data), which might affect osteogenic differentiation of pre-osteoblasts. Furthermore, one to six hours of incubation after 1 h of PFSS significantly increases osteogenic differentiation of pre-osteoblasts [26]. Interestingly, we found that low PFSS decreased *Mepe* expression. This might indicate that low PFSS enhances osteogenic differentiation in pre-osteoblasts, since targeted disruption of Of45 (osteoblast/osteocyte factor 45 gene) has been shown to enhance bone formation [8]. This assumption is consistent with findings by others, showing that the ablation of *Mepe* in null mouse leads to an increased bone mass due to increased osteoblast number and activity [8]. Additionally, osteopontin (OPN), as another SIBLING family member, is also involved in mineral regulation in the extracellular matrix of bone and dentin [58]. Both MEPE and OPN contain an acidic serine- and aspartate-rich motif (ASARM), and share 60% homology in their ASARM motifs [58]. In this study, we investigated *Opn* gene expression, which was not affected by low or high PFSS (see Appendix A (Figure A1)). Therefore, the signal of low PFSS might be mainly sensed by the integrin β subunit via a decrease in *Mepe* gene expression. Srinivasan, et al. has shown that low-magnitude loading with 10 sec of rest between each load cycle significantly increases the osteogenic potential of the regimen [59]. This supports our findings that osteoblasts might be more sensitive to low PFSS. A possible mechanism relating *Mepe* gene expression to integrin α and β expression without/with low and high PFSS is provided by the focal adhesion kinases. The cells receive different mechanical signals, and might form different focal adhesion kinases which directly connect with integrin α and β, and then with extracellular matrix protein (e.g., Mepe). On the other hand, the focal adhesion kinases directly or indirectly connect with the cytoskeleton and the nucleus, resulting in changes in cell function, e.g., Mepe gene or protein expression.

This study has some limitations. First, the integrin staining protocol is designed only for the integrin α subunit, but not for the β subunit. Future studies should also perform integrin staining for the β subunit, since it might be more sensitive to low PFSS. Second, we did not investigate the exact (mechanistic) relationship between integrin and *Mepe* expression in a bone cell under mechanical loading. Future work should investigate the mechanism of interaction between integrins and *Mepe*, using for example, silencing or blockage of integrin α or β subunits. Third, knock-out mice in the integrin α or β subunit might be a good model to validate the relationship between integrins and *Mepe* under low- and high-PFSS environments.

## 5. Conclusions

In this study, both low and high PFSS affected integrin α and β subunit expression in pre-osteoblasts, while the integrin β subunit was more altered by low PFSS. Importantly, *Mepe* gene expression was only affected by low PFSS. These results might explain the different ways *Mepe*-induced changes in pre-osteoblast mechanosensitivity may drive signaling pathways of bone cell function at low or high impact loading. These findings might have physiological and biomedical implications and require future research specifically addressing the precise role of integrin α or β subunits and *Mepe* during dynamic loading in bone health and disease.

## Data Availability

The data presented in this study are available at https://figshare.com/s/d888f330f2ab06328f70 accessed on 18 September 2024 (DOI: 10.6084/m9.figshare.27054562).
